# Relationship between symptom impairment and treatment outcome in children and adolescents with attention-deficit/hyperactivity disorder: a physician perspective

**DOI:** 10.1007/s12402-014-0143-0

**Published:** 2014-08-23

**Authors:** Juliana Setyawan, Moshe Fridman, Paul Hodgkins, Javier Quintero, M. Haim Erder, Božena J. Katić, Valerie Harpin

**Affiliations:** 1 Shire, 725 Chesterbrook Boulevard, Wayne, PA 19087 USA; 2 AMF Consulting, Inc., Los Angeles, CA USA; 3 Hospital Universitario Infanta Leonor, Madrid, Spain; 4 Ryegate Children’s Centre, Sheffield, UK; 5 Present Address: Vertex Pharmaceuticals, Boston, MA USA

**Keywords:** ADHD, Children and adolescents, Treatment outcomes, Treatment satisfaction, Symptom impairment, Comorbidities

## Abstract

We evaluated the association between those symptoms/behaviours of attention-deficit/hyperactivity disorder (ADHD) that were present at diagnosis and outcomes of treatment in children and adolescents in six European countries. Physicians abstracted clinical records from patients (6–17 years) diagnosed with ADHD between 2004 and 2007 and treated for ≥2 years. Physicians scored the severity of impairment for core ADHD symptoms and additional (non-core) ADHD symptoms/behaviours at diagnosis and estimated treatment adherence (defined as an estimated >80 % adherence on weekdays and >50 % adherence on weekends). Treatment modalities included pharmacological treatment, behavioural therapy, or both. Pharmacological treatment was further subclassified by medication class. The outcome, optimal treatment success (OTS), was defined as complete symptom control with high satisfaction with treatment. Multivariate logistic regression modelling examined the relationship between OTS and symptom impairment. Of 730 patients, 200 (27 %) achieved OTS. These patients were more likely to demonstrate lower impairment in non-core ADHD symptoms/behaviours and have fewer pre-existing comorbidities. They were also more likely to be adherent and engaged with treatment, with an explicit treatment goal to improve inattention/school performance. Neither core symptoms’ severity nor treatment types were associated with OTS. OTS rates were low, with patients having less impairment of non-core ADHD symptoms/behaviours and fewer comorbidities more likely to achieve OTS. Potentially modifiable factors affecting OTS were as follows: treatment adherence, treatment engagement, and a treatment goal to improve inattention/school performance. These data suggest that there may be opportunities to optimize current treatment use, and develop new treatment strategies to improve core and non-core ADHD symptoms/behaviours.

## Introduction

Attention-deficit/hyperactivity disorder (ADHD) is a neuro-developmental disorder with an estimated prevalence of 5–7 % in school-aged children worldwide (Polanczyk et al. 2007; Willcutt 2012), which is consistent with individual European studies (Faraone et al. 2003; Ford et al. 2003; Lecendreux et al. 2011). There is a limited understanding of the multifactorial aetiology of ADHD (Banaschewski et al. 2010; Curatolo et al. 2010; Pliszka 2007), and this is reflected in differences in diagnostic criteria (Diagnostic and Statistical Manual of Mental Disorders [DSM] [Fourth Edition] versus International Statistical Classification of Diseases [10th Revision]) and US and European management guidelines, which have been refined over time (Martenyi et al. 2009; Seixas et al. 2012; Subcommittee on ADHD 2011). ADHD commonly affects school-aged children and often persists into adulthood (Banaschewski et al. 2010; Biederman and Faraone 2005).

Core symptoms include inattention, hyperactivity, and impulsivity at levels that are significantly higher than expected in a child of that age and developmental level. However, ADHD is a heterogeneous disorder and patient presentations are wide-ranging and often complex; they frequently include the presence of additional symptoms/behaviours such as problems interacting or communicating with peers, conduct behaviours, and emotional symptoms (Becker et al. 2011), or comorbid diagnoses such as autism spectrum disorder, Tourette syndrome, depression, sleep disorder, anxiety, aggression, oppositional defiant disorder (ODD), bipolar disorder, schizophrenia, or drug abuse (Biederman et al. 1998; Rommelse et al. 2009; Spencer et al. 1999). ADHD is associated with at least one other DSM diagnosis in a vast majority of cases, affecting as many as two in three of all individuals with ADHD in the general population (Gillberg et al. 2004; Hodgkins et al. 2013; Pliszka 2007; Taurines et al. 2010).

Treatment adherence and engagement of the patient and family are positive predictors of treatment outcomes (Charach et al. 2004; Corkum et al. 1999; Gau et al. 2006; Hodgkins et al. 2011; Kaiser et al. 2008; MTA Cooperative Group 1999; Pappadopulos et al. 2009; Pierce 2011; Sanchez et al. 2005; Swanson 2003). However, several studies have suggested that the presence of additional symptoms/behaviours and/or comorbidities is associated with poorer outcomes (Becker et al. 2011; Danckaerts et al. 2010). Becker et al. (2011) found no association between core impairment levels and outcome success, and a significant association between non-core ADHD impairment levels and comorbidity with lower treatment success in children and adolescents with ADHD in standard practices in Germany. Despite the adverse impact of additional symptoms and comorbidities on treatment outcomes, the medical literature to date has provided little guidance regarding expectations and strategies for improving treatment outcomes in these more complex cases.

We conducted a retrospective cohort study that builds on a previous descriptive analysis of ADHD patient characteristics and treatment patterns in children and adolescents in six countries in the European Union (Hodgkins et al. 2013), and a sub-analysis of those who were treatment adherent (Setyawan et al. 2013). The purpose of the current study was to explore the association between the severity of ADHD symptoms/behaviours and achievement of optimal treatment success (OTS), which was defined as a combination of complete symptom control and high satisfaction with treatment as perceived by the treating physician.

## Methods

### Study design

This study was conducted as part of a retrospective chart abstraction of patient medical records by their treating physicians. Complete details regarding the study methodology can be obtained from Hodgkins et al. (2013). In brief, study participants were physicians who regularly treated patients with ADHD in France, Germany, Italy, the Netherlands, Spain, and the UK. Participating physicians identified up to five of their most recent patients who met the following criteria: (a) received a diagnosis of ADHD between January 2004 and June 2007; (b) were followed for at least 2 years after being diagnosed; (c) received either pharmacological treatment or behavioural therapy (BT) or both following the diagnosis; and (d) were not enrolled in a clinical trial during the study period. Data regarding both physician and patient characteristics were collected at the time of chart abstraction. Physicians were nominally compensated for their time.

### Outcome variable

The binary outcome variable for this study, OTS, was created using a combination of the physician’s assessment of the patient’s ADHD symptom control (completely, moderately, poorly, or not controlled) and their satisfaction level with treatment (very satisfied, moderately satisfied, neither satisfied nor dissatisfied, moderately dissatisfied, and very dissatisfied). Descriptive results from physician scoring of satisfaction with treatment demonstrated that physicians were moderately or very dissatisfied with treatment in only 3 % of patients. Given the small numbers of patients in these groups, these levels of satisfaction were combined with the ‘moderately satisfied’ and ‘neither satisfied nor dissatisfied’ responses (55 %) and compared to the ‘very satisfied’ level. Similarly, physicians reported that symptoms were not controlled in less than 0.5 % of patients and poorly controlled in 7 %, and therefore, these levels were combined with the ‘moderately controlled’ responses (62 %) and compared to the ‘complete control’ group. More details and analysis supporting the OTS definition were provided in a prior publication from this study (Setyawan et al. 2013).

### Explanatory variables

The principal explanatory variables were 12 ADHD symptoms/behaviours documented at the time of diagnosis. These included the three ‘core’ ADHD symptoms (i.e. inattention, hyperactivity, and impulsivity) (Hazell et al. 2011; Zhang et al. 2005) and additional ‘non-core’ ADHD symptoms/behaviours (categorized as anger, irritability, active defiance of reasonable requests or rules [i.e. active defiance], tendency to blame other people, challenges with school performance, social problems when interacting with family/teachers/peers/colleagues [i.e. social interaction problems], difficulty making the right choices, inappropriate behaviour, and ‘other’ symptoms/behaviours). These additional symptoms/behaviours are well known to clinicians treating ADHD, as similar items are included in assessment tools often used among the school-aged ADHD population (Goodman 1997; Swanson 1992). These additional ADHD symptoms/behaviours will be referred to as ‘non-core symptoms’. Physicians reported the presence or absence of each symptom/behaviour at diagnosis and then scored each of the core and non-core symptoms with respect to ADHD impairment using a scale from 1 to 10, with 1 being the lowest impairment and 10 being the highest impairment. Aggregated impairment scores at diagnosis were also examined for all symptoms and separately for core and non-core symptom categories.

Other clinical characteristics used as explanatory variables included: ADHD in the family (i.e. parent, sibling, or not known), comorbid diagnoses (categorized as depression, anxiety, aggression, ODD, obsessive compulsive disorder [OCD], insomnia/sleep disturbances, behavioural disorder, learning disability/difficulty, Tourette syndrome/tic disorder, epilepsy, bipolar disorder, schizophrenia, drug abuse, alcohol abuse, and autism spectrum disorder), and the total number of comorbid conditions present at diagnosis.

The study definition of initiating a new ‘therapy’ was either the addition or discontinuation of an ADHD medication or BT. For each patient, the most recent five therapies were abstracted. For this study, the analysis was based on the ‘current’ therapy (i.e. that which was currently being taken by the patient at the time of chart abstraction).

Treatment was categorized based on two different rules: treatment modality and treatment type. Treatment modality included pharmacological therapy only, BT only, or both. In this study, there was no differentiation of type of BT (child, parent, and family). Treatment type included all three modalities and was further refined by subclassifying the pharmacological treatment group by medication classes: long-acting (LA) methylphenidate (MPH), short-acting (SA) MPH, SA amphetamine, atomoxetine, other pharmacotherapy, and multiple pharmacotherapies. Additional explanatory variables related to treatment included the number of therapies recorded on the patient’s chart (up to the most recent five), the number of years of follow-up since diagnosis, the number of therapies per follow-up year, and concomitant psychotropic medications.

The following pre-specified treatment goals were also reviewed and analysed: improve concentration/functioning at school/work (i.e. improve inattention), control hyperactivity, control aggression, control impulsivity, increase self-esteem, reduce chances of substance abuse, enable patient to build relationships, enable patient to maintain relationships, improve behaviour, reduce likelihood of being in trouble, reduce disruption at home, enable participation in activities outside of school, minimize chance of exclusion from school/work, improve family relationship, and other. Multiple answers were allowed per patient. Goals were grouped based on clinical considerations and empirical evidence (i.e. factor analysis) to reduce the number of categories. As treatment goals represent expectations from treatment and may be associated with physician-reported satisfaction with treatment and symptom control, the grouped goals were included as potential predictors.

Patient engagement with treatment and family involvement with treatment were measured independently and continuously on a scale from 1 to 10, with 1 being ‘no engagement/involvement’ and 10 being ‘strong engagement/involvement’.

A patient was considered adherent to pharmacotherapy when the physician reported that he or she was believed to be taking the medication at least 80 % of the time on weekdays and 50 % on weekends and holidays. Adherence was also defined for BT (i.e. 80 % of scheduled sessions), and if BT did not take place on weekends or holidays, then only the weekday value was used for classification.

### Statistical analyses

Descriptive statistics were reported for the study population, and the identification of predominant ADHD symptoms/behaviours at diagnosis and mean impairment scores were compared by OTS. Descriptive statistics included: the frequency (*n*), the percentage (%), the mean, standard deviation (SD), median, and interquartile range. Deviations from expected rates in categorical variables were tested with one degree of freedom chi-square tests. Covariates were tested individually to assess the significance of their association with OTS (bivariate two-sample t tests and chi-square tests for continuous and categorical covariates, respectively).

Multiple logistic regression was used to examine the relationship between OTS and the level of symptom/behaviour impairment, adjusted for other covariates that were significantly associated with OTS in bivariate tests. Covariates significantly associated with the outcome (*p* < 0.05) were included in a stepwise multiple logistic regression (*p* < 0.05 for entry and retention) to select a subset of simultaneously significant covariates that were associated with OTS. Odds ratios (ORs) with 95 % confidence intervals (CIs) were reported for the final selected model.

Finally, after the significant main effects were selected with the stepwise procedure, second-order terms (interactions and squared continuous covariates) were tested for the selected model, and those significant over and above the main effects were retained. The Hosmer–Lemeshow goodness-of-fit test was used to assess the adequacy of the model, and the *c*-statistic was used to evaluate the accuracy of prediction. The *c*-statistic ranges from 0.5 to 1, where *c* = 1 for a perfect model and *c* = 0.5 for a model no better than random classification (Hosmer and Lemeshow 1989). To illustrate the relationships explained by the final model, curves describing the estimated probability of OTS were calculated for several combinations of covariates.

To provide further interpretability for the model ORs, multiple logistic regression modelling was repeated using categorical variables for continuous predictors of OTS. The aggregate non-core symptom impairment variable was replaced with the categorical variable ‘3 or more non-core symptoms (Y/N)’, and the level of patient engagement was replaced with dummy variables indicating the third and fourth quartiles of the patient engagement score (using the lower 50 % of engagement scores as a reference).

All reported tests were two-sided at a significance level of *α* = 0.05 significance level. Data were analysed using SAS statistical software (version 9.2, SAS Institute, Inc., Cary, NC, USA). This study complied with all US and International Conference on Harmonization human subjects ethics committee requirements and was approved by the Research Triangle Institute institutional review board.

## Results

Data were collected by 340 physicians from 779 eligible patients. Of these 779 patients, 730 were receiving ADHD medication and/or BT at the time of the chart review and were included in this analysis. This final study population had a mean (SD) age of 12.0 (2.6) years and 82 % were aged 10–17 years (range 6–17 years); the majority were male (77 %). Almost one-third (28 %) had an immediate family member diagnosed with ADHD. The distribution of patients (physicians) by country was as follows: France 118 (50), Germany 137 (52), Italy 134 (73), the Netherlands 72 (55), Spain 132 (50), and the UK 137 (57). Overall, 48.5 % of patients were treated by psychiatrists (France 88.5 %, Germany 36.4 %, Italy 9.7 %, the Netherlands 50.0 %, Spain 66.2 %, and the UK 56.8 %).

Of all patients, 30 % had two core ADHD symptoms and 44 % had all three core ADHD symptoms of inattention, hyperactivity, and impulsivity. For non-core symptoms, fewer than 5 % had none and 64 % had three or more. Forty-three per cent had all three core symptoms plus between five and eight of the non-core symptoms, suggesting severe difficulties. The mean (SD) total impairment score for all twelve symptom/behaviour impairment scores combined was 6.5 (1.5), whereas the mean (SD) impairment scores for the three core symptoms and for the nine additional non-core symptoms were 7.3 (1.6) and 6.2 (1.7), respectively.

Characteristics of the study population by OTS are described in Table 1. Overall, physicians reported OTS in 27 % (200/730) of patients. Among patients achieving OTS, 56 % had three or more additional non-core symptoms compared with 66 % among those not achieving OTS (*p* = 0.009) (Table 1). There was no significant difference in the mean core symptoms impairment score between those who did and did not achieve OTS (7.2 vs 7.4, *p* = 0.190), but those who achieved OTS had a significantly lower mean impairment score for the non-core symptoms (5.7 vs 6.4, *p* < 0.0001).

**Table 1:** Patient and clinical characteristics associated with optimal treatment success

	Optimal treatment success^a^ (*n* = 200)	Non-optimal treatment success^a^ (*n* = 530)	*p* value^b^
Three or more non-core symptoms, n (%)	112 (56.0)	352 (66.4)	**0.009**
ADHD symptomatic average impairment level—all symptoms			**<0.0001**
Mean (SD)	6.10 (1.61)	6.66 (1.41)	
Median (range)	6.1 (2.5–10.0)	6.8 (1.8–10.0)	
ADHD symptomatic average impairment level—core symptoms			0.190
Mean (SD)	7.21 (1.71)	7.39 (1.49)	
Median (range)	7.3 (2.0–10.0)	7.7 (3.0–10.0)	
ADHD symptomatic average impairment level—non-core symptoms			**<0.0001**
Mean (SD)	5.68 (1.79)	6.39 (1.56)	
Median (range)	5.8 (1.6–10.0)	6.6 (1.0–10.0)	
Number of pre-existing comorbidities			**<0.0001**
Mean (SD)	2.09 (1.85)	3.00 (2.11)	
Median (range)	2.0 (0.0–7.0)	3.0 (0.0–9.0)	
Male sex, *n* (%)	145 (73.5)	420 (79.3)	0.059
Patient engagement^c^			
Mean (SD)	7.45 (1.59)	5.92 (2.09)	
Median (range)	8.0 (2.0–10.0)	6.0 (1.0–10.0)	**<0.0001**
Family involvement^d^			
Mean (SD)	8.46 (1.29)	7.51 (1.79)	**<0.0001**
Median (range)	9.0 (3.0–10.0)	8.0 (1.0–10.0)	
Treatment goals (multiple per patient), *n* (%)			
Restrain inappropriate behaviour (factor)	114 (57.0)	360 (67.9)	**0.0070**
Control hyperactivity	158 (79.0)	452 (85.3)	**0.0443**
Improve inattention	179 (89.5)	390 (73.6)	**<0.0001**
Treatment adherent^e^	170 (85.4)	335 (65.8)	**<0.0001**

*ADHD* attention-deficit/hyperactivity disorder, *SD* standard deviation

^a^Percentage for categorical variables and mean (SD) for continuous variables

^b^Significant chi-square *p* values (*p* < 0.05) in bold

^c^Physician-rated extent of patient engagement in ADHD condition and treatment (1 = no engagement and 10 = strong engagement)

^d^Physician-rated involvement of family/caregiver in patient’s ADHD condition and treatment (1 = no involvement and 10 = strong involvement)

^e^22/730 patients were missing adherence data. Adherence was defined as taking the treatment for at least 80 % of the time on weekdays and 50 % on weekends and holidays

Individuals who achieved OTS were perceived to have higher average patient engagement (7.5 vs 5.9, *p* < 0.0001) and family involvement levels (8.5 vs 7.5, *p* < 0.0001). Of the patients who achieved OTS, 57.0 % had restraint of inappropriate behaviour as a treatment goal (factor) compared with 67.9 % of those who did not achieve OTS (*p* = 0.0070). The restraint of inappropriate behaviour as a treatment goal factor included the following individual treatment goals: control aggression, reduce chances of substance abuse, reduce likelihood of being in trouble, and minimize chance of exclusion from school/work. A similar pattern was observed for control of hyperactivity as a treatment goal (*p* = 0.0443), whereas the pattern was reversed for improving attention as a treatment goal—of those who achieved OTS, 90 % had improving inattention as a treatment goal compared with 74 % of patients who did not achieve OTS (*p* < 0.0001).

Physicians reported that 71 % of the patients were considered to be adherent to the ADHD treatment, with patients achieving OTS more likely to be adherent (85 vs 66 %, *p* < 0.0001), or conversely stated, adherent patients were more likely to achieve OTS (33.7 % adherent and 14.3 % non-adherent). Fifty-three per cent of the patients received the same treatment type over the entire study duration for a mean (SD) of 2.5 (1.2) years, and there was no difference in the number of therapies by OTS group. At the time of data abstraction, most patients (66 %; 483/730) were receiving MPH; few (1 %; 9/730) were receiving amphetamines (data not shown).

**Fig. 1:**
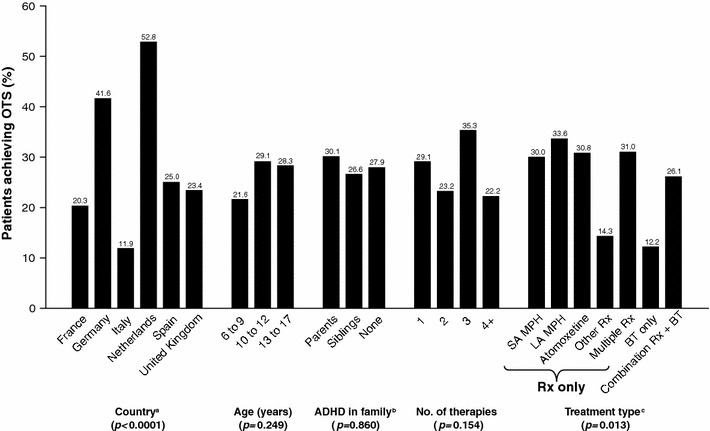
Characteristics associated with optimal treatment success. ^a^ Germany (*p* = 0.002) and the Netherlands (*p* < 0.0001) had a significantly higher OTS rate and Italy (*p* < 0.001) a significantly lower OTS rate compared to the overall OTS rate. ^b^ *n* = 668 due to missing values. ^c^ ‘BT only’ was the only treatment type with a significantly different OTS rate compared to the overall OTS rate (*p* = 0.006). *BT* behavioural therapy, *LA* long acting, *MPH* methylphenidate, *Rx* pharmacotherapy, *SA* short acting. ‘Other Rx’ included medications other than MPH, amphetamine, and atomoxetine; ‘No. of therapies’ denotes number of therapies (as per study definition) recorded on the patient’s chart

There were significant differences in OTS rates by country (Fig. 1), with higher than average rates of 53 % (38/72) in the Netherlands (*p* < 0.0001) and 42 % (57/137) in Germany (*p* = 0.002) and a lower than average rate of 12 % (16/134) in Italy (*p* < 0.001). Other notable differences across countries included: (1) The Diagnostic and Statistical Manual of Mental Disorders 4th edition diagnostic approach was most commonly used in the Netherlands (81.1 %) and Spain (79.1 %), while the International Classification of Diseases 10th Revision was most common in Germany (87.4 %); (2) the UK had the highest rate of treatment with pharmacotherapy alone (63.7 %), while treatment with BT alone was most common in Italy (41.0 %). Over 54 % of patients in Italy used SA MPHs, while Spain did not report any use. Atomoxetine was most commonly used in Italy (36.7 %), but it was not available in France. SA amphetamines were not used in France or Spain, were used by less than 2 % of patients in Germany, the UK, and the Netherlands, and were most common in Italy (9.0 %); and (3) adherence to ADHD treatment ranged from 50.8 % in Italy to 80.3 % in France.

The rate of OTS was lowest for patients receiving BT only (12 %; 11/90), and this group was the only group with a significant deviation from the overall expected 27 % OTS rate (*p* = 0.006). There was no significant difference in OTS rate for the remaining current treatment type categories (all included medications), ranging from 26 % (70/268) for the combination of pharmacotherapy + BT to 34 % (72/214) for LA MPH.

**Fig. 2:**
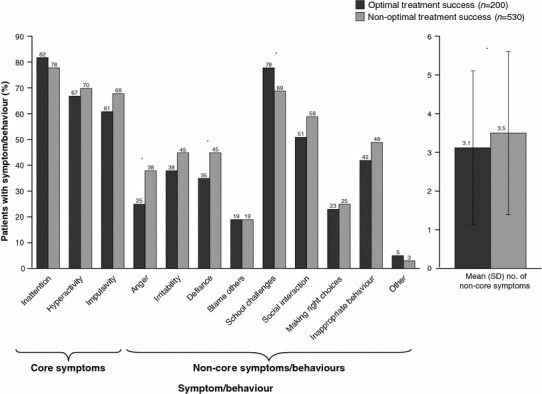
Symptoms present at ADHD diagnosis. Patients could have multiple predominant symptoms. *p < 0.05. *ADHD* attention-deficit/hyperactivity disorder, *SD* standard deviation

Figure 2 shows the percentage of patients with impairment at diagnosis across all symptoms/behaviours for those who did and did not achieve OTS. Non-OTS patients had significantly higher rates of anger (*p* = 0.002) and defiance (*p* = 0.009). Non-significant relationships were observed for all the other symptoms/behaviours, with the exception of challenges with school performance, which was significantly negatively associated with OTS (*p* = 0.022). Patients who did and did not achieve OTS presented with an average of 3.1 versus 3.5 non-core symptoms (*p* = 0.026), respectively.

**Fig. 3:**
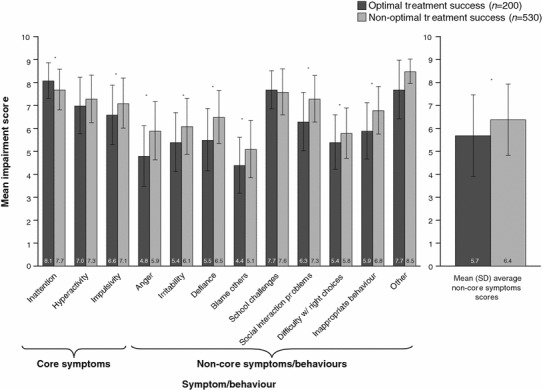
Mean (SD) impairment score at ADHD diagnosis. *p < 0.05. *ADHD* attention-deficit/hyperactivity disorder, *SD* standard deviation

Figure 3 shows the mean symptom/behaviour impairment scores for those who did and did not achieve OTS. For non-OTS patients, a significantly higher mean impairment score was observed for the individual symptoms of impulsivity (*p* = 0.005), anger (*p* < 0.0001), irritability (*p* < 0.001), defiance (*p* < 0.0001), tendency to blame other people (*p* = 0.001), social interaction problems (*p* < 0.0001), difficulty making the right choices (*p* = 0.025), and inappropriate behaviour (*p* < 0.0001). Inattention was the only symptom for which the impairment score was significantly higher in those with OTS (*p* = 0.018).

**Fig. 4:**
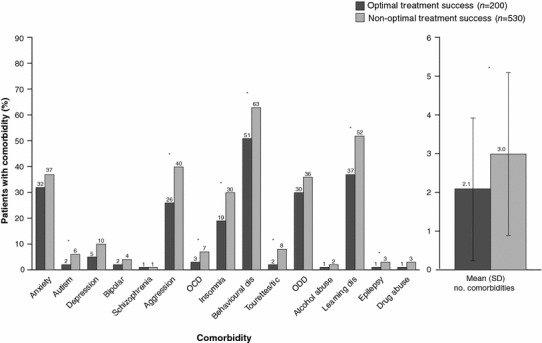
Psychiatric comorbidities present at ADHD diagnosis. *p < 0.05. *ADHD* attention-deficit/hyperactivity disorder, *Behavioural Dis* behavioural disturbances, *Learning Dis* learning disabilities, *OCD* obsessive compulsive disorder, *ODD* oppositional defiance disorder, *SD* standard deviation

Most of the patients (77 %) had at least one psychiatric or developmental comorbidity. Figure 4 describes the proportion of patients with each of the documented comorbidities by OTS group. The mean number of pre-existing comorbidities was significantly lower in the patients who experienced OTS (2.1 vs 3.0, *p* < 0.0001). Non-OTS patients were more likely to present with autism spectrum disorder (*p* = 0.015), aggression (*p* < 0.0001), OCD (*p* = 0.029), insomnia/sleep disturbances (*p* = 0.001), behavioural disorder (*p* = 0.005), Tourette syndrome/tic disorder (*p* < 0.001), learning disabilities (*p* < 0.001), and epilepsy (*p* = 0.033) compared with patients who achieved OTS.

The final multivariate logistic regression model is described in Table 2. For this model, the impairment scores for non-core symptoms were aggregated into a single variable. This variable did not include challenges with school performance or other symptoms, as these individual symptoms/behaviours were not statistically associated with OTS. A lower non-core symptom impairment score was highly associated with OTS. Patients who had a lower non-core symptom impairment level (i.e. 1 SD or 1.8 points lower) were 1.31-fold more likely to achieve OTS (the OR from the model was 0.762, and the reversed OR for the reverse interpretation of the predictor was 1/0.762). Covariates that significantly contributed to the model for OTS were as follows: treatment adherence (OR 2.0), fewer comorbidities [specifically no pre-existing autism spectrum disorder (reversed OR 4.4) and no Tourette syndrome/tic disorder (reversed OR 4.4)], higher patient engagement [OR 3.4 for 1 SD (i.e. 2.1 points higher) for a reference patient in Germany], the treatment goal to improve attention/school performance (OR 1.8), the country of residence, and an interaction term between patient engagement and country. The Netherlands had a significantly higher OTS rate compared with Germany, and the UK and France had a significantly lower OTS rate (results reported for a median patient engagement level of 7). The adjusted effect of treatment type was not statistically significant.

**Table 2:** Multiple logistic regression model for predicting OTS

Covariate	OR (95 % CI) *c* = 0.80^a^ *p* = 0.496^b^
Non-core symptoms: ADHD impairment	0.762 (0.621, 0.934)
Average (1–10) [mean (SD) = 6.0 (1.8)]^c^	
Pre-existing autism	0.229 (0.063, 0.835)
Pre-existing Tourette syndrome/tic disorder	0.229 (0.064, 0.828)
Treatment adherence^d^	2.025 (1.235, 3.319)
Improved attention treatment goal	1.790 (1.025, 3.125)
Patient engagement^e^ (1–10) [mean (SD) = 6.4 (2.1)]	3.390 (1.829, 6.285)
Country (Germany as reference)^f^	
France	0.461 (0.248, 0.857)
Italy	0.588 (0.281, 1.229)
Netherlands	2.232 (1.129, 4.425)
Spain	0.664 (0.368, 1.198)
UK	0.399 (0.189, 0.845)

*ADHD* attention-deficit/hyperactivity disorder, *CI* confidence interval, *OR* odds ratio, *OTS* optimal treatment success, *SD* standard deviation

^a^ *c*-statistic of 1 indicates a perfect model, and *c*-statistic of 0.5 indicates the model is no better than random classification

^b^Hosmer–Lemeshow test

^c^Defined as the non-core ADHD symptom impairment average for the individually significant symptom impairments (anger, irritability, defiance, blame others, social interaction problems, difficulty making right decisions, and inappropriate behaviour). This variable did not include challenges with school performance or other symptoms, as these individual symptoms/behaviours were not statistically associated with OTS

^d^Defined as >80 % adherence on weekdays and >50 % adherence on weekends

^e^Interacted with country: OR (95 % CI) reported for reference country (Germany)

^f^Interacted with patient engagement: OR (95 % CI) reported for median patient engagement level of 7.0

The *c*-statistic for the logistic regression model was 0.80, indicating that the model correctly classified OTS for about 80 % of patients. The Hosmer–Lemeshow *p* value was 0.496, demonstrating a good fit of the model.

**Fig. 5:**
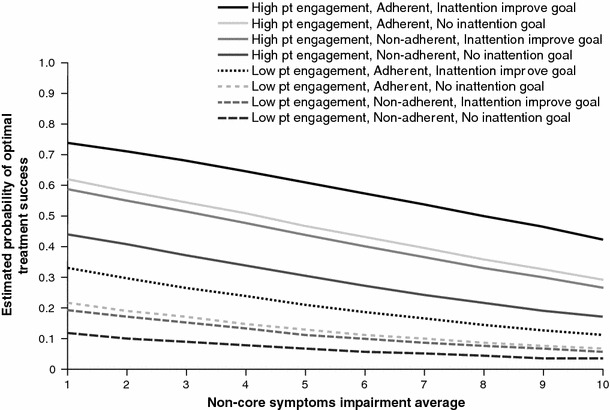
Estimated probabilities of optimal treatment success by non-core symptoms impairment average, level of patient engagement, adherence to treatment, and inattention improvement as treatment goal. Estimated probabilities from multiple logistic regression model for patients from Germany (as the reference country) not presenting with autism or Tourette syndrome. High and low patient engagement levels were defined as 5 (25th percentile) and 8 (75th percentile), respectively. *Pt* patient

Figure 5 illustrates the model outcome, OTS, by the aggregated non-core symptom impairment score for eight examples of hypothetical patients based on model predictor combinations. This figure can be used to estimate the probability of OTS by average non-core symptom impairment level, given different combinations of patient engagement levels, adherence to treatment, and the treatment goal to improve attention. Patients in Germany who did not present with autism spectrum disorder or Tourette syndrome/tic disorder at diagnosis were used as representative fixed values for all sample-estimated probability curves, and low and high patient engagement levels were fixed at 5 (25th percentile) and 8 (75th percentile), respectively.

As expected, the highest probability of OTS for each fixed level of patient engagement was predicted to be achieved by patients with a lower average non-core symptom score who were adherent to treatment and had improving inattention/school performance as a treatment goal. Conversely, patients with the lowest probability for achieving OTS were those who, at each fixed level of patient engagement, demonstrated a higher average impairment score for non-core ADHD symptoms, were not adherent to treatment, and did not have ‘improving inattention/school performance’ as a treatment goal at diagnosis. As indicated by the relative magnitude of the estimated ORs, patient engagement had the highest effect size among the predictors allowed to vary in Fig. 5, followed by adherence, treatment goal to improve inattention/school performance, and average non-core symptom impairment level. For example, an adherent patient with an engagement score of 8, an average non-core symptom impairment score of 2, and without improvement in inattention/school performance as a treatment goal is predicted to have a 58.3 % (95 % CI, 38.9, 75.4 %) estimated probability of OTS versus 50.8 % (95 % CI, 34.1, 67.4 %) and 43.3 % (95 % CI, 28.3, 59.8 %) for similar patients with average impairment scores for non-core symptoms of 4 and 6, respectively.

For improved interpretability, a separate logistic regression model was developed that replaced continuous covariates of non-core symptom impairment and level of patient engagement with the categorical variables as defined in the Methods section. The OR for the categorical predictor ‘less than 3 non-core symptoms’ was 1.61 (95 % CI, 1.10, 2.35), indicating that patients with two or fewer non-core symptoms were 61 % more likely to achieve OTS than patients with three or more (maximum seven) non-core symptoms. For patients with an engagement score in the third or fourth quartiles, the engagement ORs relative to patients in the first and second engagement score quartiles were 2.16 (95 % CI, 1.29, 3.60) and 4.46 (95 % CI, 2.86, 6.95), respectively. Estimated ORs for the other covariates changed only slightly (data not shown).

## Discussion

This study used physician-reported data to analyse the association between ADHD symptom impairment at diagnosis and treatment outcomes to gain a perspective on the relative effect of symptom impairment, comorbid psychiatric conditions, and other patient factors on OTS in a European routine clinical care setting. Our results showed that the physician’s assessment of treatment success was associated with both the presence and the level of impairment of non-core ADHD symptoms/behaviours at diagnosis and was not associated with core symptoms, suggesting that current ADHD treatments may be most effective for core symptoms in comparison to non-core symptoms and comorbidities. A lower average impairment score of these non-core symptoms was associated with a higher likelihood of attaining OTS in our models.

In addition to the presence and severity of non-core symptoms, several other predictors affected OTS. The comorbidities of autism spectrum disorder or Tourette syndrome/tic disorder were independently associated with a decreased OTS rate.

Although data regarding treatment success for these complex patients under routine care are scarce, our results are consistent with other studies demonstrating that as the severity of the disorder increases or becomes complicated by comorbidity or psychosocial stressors, health-related quality of life (HRQoL) impairment also worsens (Danckaerts et al. 2010; Gillberg et al. 2004). Whereas patients and family members usually assess patient HRQoL, clinicians often assess patient symptom severity and functional impairment, and these aspects have been also linked to decreased HRQoL in patients with ADHD (Danckaerts et al. 2010; Sawyer et al. 2002).

Although the specific type of treatment did not appear to be associated with OTS, other potentially mutable factors affecting OTS were identified by this study, including adherence to treatment, patient engagement with treatment, and the presence of a treatment goal to improve inattention/school performance. These factors have all been hypothesized and supported by previous literature. The effectiveness of treatment, particularly pharmacotherapy, has been well established (Pliszka 2007), and adherence to treatment has been shown to improve outcomes not only of ADHD symptoms (MTA Cooperative Group 1999), but also of measures of maternal and family functionality (Gau et al. 2006). Conversely, limited patient and family adherence to pharmacotherapy and BTs have been identified as barriers to maximizing the effects of ADHD treatment (Charach et al. 2004; Corkum et al. 1999; Hodgkins et al. 2011; Kaiser et al. 2008; Pappadopulos et al. 2009; Pierce 2011; Sanchez et al. 2005; Swanson 2003). As a core symptom, inattention has been shown to improve with pharmacological treatment (MTA Cooperative Group 1999), and clear goals and strategies to reduce inattention may have led to well-defined expectations among treating physicians to recognize improvement in this area. Other non-core symptoms, particularly behavioural symptoms, are less well known to respond to pharmacological treatment, and fewer benchmarks have been defined to measure improvement in these other symptoms (Gillberg et al. 2004; Kaiser et al. 2008).

Treatment options in some parts of Europe are limited and have varied over the time of the study, given that patients primarily have access to BT, MPH, and atomoxetine, individually or in combination. In this study, there were no significant differences in OTS rates among treatment types, perhaps due to the limited pharmacological options available. On the other hand, adherence to (any) treatment doubled the patient’s odds of achieving OTS. Given the unsatisfactory rate of OTS in the overall population (27 %), improved outcomes may be achievable by optimizing the conditions shown to affect success. Physicians and patients/caregivers should consider engaging in dialogue to ensure treatment goals are being met. In particular, efforts should be made to engage patients and their families as much as possible in their treatment (as engagement is a strong predictor of OTS) and to improve treatment adherence. Where treatment goals are not achieved, alternative treatment options may be considered. Additionally, the development of new pharmacological therapy and BT strategies for the improvement of core and non-core symptoms, particularly for use in patients with complex comorbidities, appears warranted.

Further research is needed to investigate observed variations in the rates of OTS by country. The availability of different medications and resources such as family and community support or supportive educational settings, differences in physician training and practice setting across countries, possible differences in physician perception of control, treatment priorities, national standards, insurance affordability, and cultural atmosphere all vary by country (Curatolo et al. 2010; Martenyi et al. 2009; Schlander et al. 2007; Seixas et al. 2012). This study was not designed to address the reasons for these differences.

There are several limitations to this study that deserve mention. Although this was a large observational study relative to other published studies in the field, the generalizability of these results at the population or country levels remains limited because of its reliance on a convenience sample. However, although absolute rates of OTS and predictor values may have been limited by the nature of the study sample, the results obtained from ORs (generated by logistic regression modelling) should be minimally affected as OR estimates are independent of the sampling design.

The OTS outcome measure was a new composite measure developed for this study and was derived from measures of physician-reported symptom control and satisfaction with treatment. Satisfaction measures are often skewed towards higher levels of satisfaction and frequently require categorization of results to achieve interpretability (Williams et al. 1998). Simplification of the analysis by focusing on the patients with the best outcomes likely underestimated the rate of treatment success. However, given that the goal of ADHD management is to strive for optimal, rather than intermediate, outcome and given that moderate and poor outcome groups showed greater similarity to each other in their relationship with ADHD symptoms, our definition of OTS appeared adequately supported by the data at hand. Furthermore, this approach is analogous to accepted dichotomization of response defined by the Clinical Global Impression scale into 1 or 2 (response) versus 3 or greater (non-response), which also underestimates treatment response. Similarly, having responders defined by a threshold of 25 %, as commonly defined in clinical papers, is also somewhat arbitrary and underestimates treatment response.

This study relied on physician-reported data, which may not be entirely consistent with the perception of the patients or their carers. If these data had been collected from various independent sources, particularly with respect to OTS, it is possible that correlations observed in this study may have been attenuated. The source of information in the charts might vary, and ADHD impact and symptom ratings can vary by informant. Data on the informant were not collected and did not account for this potential source of variation. Because our OTS measure has not been psychometrically validated, there is no estimate of the variability attributed to test–retest discordance and to differences in interpretation of satisfaction and symptom control across physicians and countries. Additionally, the definitions and rating scales for the non-core ADHD symptoms had not been validated prior to their use in this investigation. In some cases, the non-core ADHD symptoms and comorbid diagnoses used in this study overlap. Some non-core ADHD symptoms (e.g. active defiance or inappropriate behaviour) constitute core symptoms for comorbid conditions (e.g. ODD or behavioural disorder), and it can be argued that there is no clear distinction between these two domains of covariates. However, the multiple regression model presented contains an optimal selection of covariates chosen from the complete pool of covariates that represented a comprehensive measurement of impairment and comorbid burden. Retrospective evaluation of treatment goals and presence and severity of symptoms by physicians may have been biased. To try to minimize the bias, physicians were instructed in the questionnaire to rely solely on chart information to respond to questions rather than on memory. The results of this study should be confirmed by future studies using more targeted investigations that employ defined sampling schemes, patient- and carer-reported adherence and symptom control measures, and comprehensive outcome measures with established validity. Ideally, studies should utilize population-based cohorts.

## Conclusion

This study provides insight regarding the impact of a wide range of ADHD symptoms and behaviours on treatment outcomes for children and adolescents under routine clinical care as documented by their physicians. Overall, physician-reported OTS in this European routine care setting seems to be low. High impairment levels in non-core symptoms were negatively associated with OTS, whereas impairment levels in core ADHD symptoms were not. Future research is necessary to confirm and further understand this observation. Potentially mutable factors associated with OTS included treatment adherence, patient engagement, and specific goals of improving symptoms of inattention/school performance. Achieving OTS is likely to be more challenging for patients with comorbid conditions, and this should be taken into consideration by physicians and discussed with the patients and their families.

The variability and complexity of the presentation of patients with ADHD that is highlighted here, coupled with the apparently low treatment success rate, suggest that opportunities exist for the development of improved treatments and support for children and young people with ADHD and their families.
